# Anthocyanins from *Hibiscus syriacus* L. Inhibit Melanogenesis by Activating the ERK Signaling Pathway

**DOI:** 10.3390/biom9110645

**Published:** 2019-10-24

**Authors:** Wisurumuni Arachchilage Hasitha Maduranga Karunarathne, Ilandarage Menu Neelaka Molagoda, Sang Rul Park, Jeong Woon Kim, Oh-Kyu Lee, Hae Yun Kwon, Matan Oren, Yung Hyun Choi, Hyung Won Ryu, Sei-Ryang Oh, Wol Soon Jo, Kyoung Tae Lee, Gi-Young Kim

**Affiliations:** 1 Department of Marine Life Sciences, Jeju National University, Jeju 63243, Korea; hasikarunarathne@gmail.com (W.A.H.M.K.); neelakagm2012@gmail.com (I.M.N.M.); srpark@jejunu.ac.kr (S.R.P.); 2 Forest Biomaterials Research Center, National Institute of Forest Science, Jinju 52817, Korea; jesi2000@korea.kr (J.W.K.); okaylee@korea.kr (O.-K.L.); 3 Forest Tree Improvement Division, Bioresources Research Center, National Institute of Forest Science, Suwon 1663, Korea; kwonhy05@korea.kr; 4 Department of Molecular Biology, Ariel University, Science Park, Ariel 40700, Israel; matanor@ariel.ac.il; 5 Department of Biochemistry, College of Oriental Medicine, Dong-Eui University, Busan 47227, Korea; choiyh@deu.ac.kr; 6 Natural Medicine Research Center, Korea Research Institute of Bioscience and Biotechnology, Cheongju 28116, Korea; ryuhw@kribb.re.kr (H.W.R.); seiryang@kribb.re.kr (S.-R.O.); 7 Department of Research Center, Dong Nam Institute of Radiological and Medical Sciences, Busan 46033, Korea; sailorjo@dirams.re.kr

**Keywords:** *Hibiscus syriacus* L., anthocyanin, melanin, tyrosinase, ERK

## Abstract

*Hibiscus syriacus* L. exhibited promising potential as a new source of food and colorants containing various anthocyanins. However, the function of anthocyanins from *H. syriacus* L. has not been investigated. In the current study, we evaluated whether anthocyanins from the *H. syriacus* L. varieties Pulsae and Paektanshim (PS and PTS) inhibit melanin biogenesis. B16F10 cells and zebrafish larvae were exposed to PS and PTS in the presence or absence of α-melanocyte-stimulating hormone (α-MSH), and melanin contents accompanied by its regulating genes and proteins were analyzed. PS and PTS moderately downregulated mushroom tyrosinase activity *in vitro*, but significantly decreased extracellular and intracellular melanin production in B16F10 cells, and inhibited α-MSH-induced expression of microphthalmia-associated transcription factor (MITF) and tyrosinase. PS and PTS also attenuated pigmentation in α-MSH-stimulated zebrafish larvae. Furthermore, PS and PTS activated the phosphorylation of extracellular signal-regulated kinase (ERK), whereas PD98059, a specific ERK inhibitor, completely reversed PS- and PTS-mediated anti-melanogenic activity in B16F10 cells and zebrafish larvae, which indicates that PS- and PTS-mediated anti-melanogenic activity is due to ERK activation. Moreover, chromatography data showed that PS and PTS possessed 17 identical anthocyanins as a negative regulator of ERK. These findings suggested that anthocyanins from PS and PTS inhibited melanogenesis *in vitro* and *in vivo* by activating the ERK signaling pathway.

## 1. Introduction

Melanocytes are melanin-producing neural crest-derived cells located in the basal layer of skin epidermis, and they transfer melanin to the neighboring keratinocytes to protect the cells from ultraviolet radiation (UV)-mediated cell damages [1]. Melanin is a dark pigment responsible for not only skin, eye and hair color, but also prevention of UV induced skin injuries [2]. Therefore, melanin has been thought as a major photoprotective factor against UV-induced oxidative stress and damages. However, abnormal accumulation of melanin causes dermatological problems such as melasma, wrinkling, senile lentigines and skin cancer [3,4]. In addition, interest in skin whitening agents has been greatly increasing in the cosmetic industry. In regards, many anti-melanogenic compounds targeting tyrosinase, a major rate-limiting enzyme of melanin biosynthesis, have been developed [5,6].

Melanogenesis, the physiological process of melanin production, is regulated by various molecular signaling pathways with chains of enzymatic and non-enzymatic reactions. Tyrosinase and tyrosinase-related protein-1/2 (TRP-1/2) play a crucial role in increasing melanin generation through hydroxylation of tyrosine into dihydroxyphenylalanine (DOPA), followed by further oxidation of DOPA into DOPA quinone [7]. Since tyrosinase is exclusively necessary for melanogenesis, it has been used as a target in the development of melanogenesis inhibitors. In addition, microphthalmia-associated transcription factor (MITF) is a pivotal transcription factor that upregulates the expression of tyrosinase and TRP-1/2 at the transcriptional level under UV exposure, which stimulates melanogenesis [8,9]. During melanogenesis, α-melanocyte-stimulating hormone (α-MSH), an endogenous peptide hormone, binds to the melanocortin 1 receptor (MC1R), which belongs to the G-protein receptor family, in melanocytes, thus increasing the intracellular level of cyclic adenosine 3′5′-monophosphate (cAMP) by activating adenylyl cyclase (AC) and stimulating protein kinase A (PKA) [10]. Next, cAMP-responsive element binding protein (CREB) leads to the phosphorylation and upregulation of MITF expression [11]. In contrast, previous studies revealed that extracellular signal-regulated kinase (ERK) phosphorylation inhibits melanogenesis by accelerating proteasomal degradation of MITF, which is accompanied by mitochondrial fission [12,13]. Recent studies have also found melanogenesis inhibitors that negatively regulate the cAMP-dependent pathway and positively stimulate the ERK pathway [5,6]. Moreover, the Wnt/β-catenin signaling pathway has been studied as a potential regulator of melanogenesis in relation to *MITF* transcription [14,15].

*Hibiscus syriacus* L., called by rose of Sharon, is the Korean national flower and widely distributed from Southern Asia to Northern Asia. *H. syriacus* L. has been known as a medicinal herb; its dried root and stem bark have been used as antidotes, spring tonics and fever reducers in Korean traditional remedy. Recent studies also revealed that extracts of the bark and rhizosphere of *H. syriacus* L. exert significant wound healing activity and protective activity against UV-mediated photoaging in fibroblasts and keratinocytes by stimulating collagen and fibronectin synthesis [16,17]. Moreover, new medicinal effects of *H. syriacus* L. have been elucidated, namely anti-depressant and neuroprotective [12], anti-cancer [18,19] and anti-oxidant [20] activities. Nevertheless, the flower petals of *H. syriacus* L. have not been investigated for medicinal and functional effects.

We, in the current study, investigated the effects of anthocyanins from two *H. syriacus* L. varieties, Pulsae and Paektanshim (PS and PTS, respectively) which have different petal colors (Pulsae: purple; Paektanshim: white), on melanogenesis regulation in α-MSH-treated B16F10 cells and zebrafish larvae, because B16F10 cells and zebrafish larvae have been widely used for melanin formation due to its genomic correlation with human pigmentation [21,22,23]. PS and PTS significantly downregulated melanogenesis in B16F10 cells and zebrafish larvae by inhibiting the expression of MITF and tyrosinase.

## 2. Materials and Methods

### 2.1. Extraction of PS and PTS

*H. syriacus* L. Pulsae and Paektanshim were cultivated in the *Hibiscus* clonal archive of the Korea Forest Research Institute, Suwon, Republic of Korea (N 37° 15′ 5.56″, E 126° 57′ 16.11″) between July and August 2017 and identified by Dr. H.-Y. Kwon (one of the authors). Voucher specimens were deposited in the Korea Forest Service (NF-H8-F; http://english.forest.go.kr/newkfsweb/eng/idx/Index.do?mn=ENG_01).

The petals of *H. syriacus* L. Pulsae and Paektanshim were freeze-dried for 3 days and then stored at below −20 °C before extraction. Secondary metabolites were obtained through extraction in accordance with a previously described procedure [19], with slight modification. Briefly, the petals (1.5 kg) were ground, extracted three times with 95% ethanol (40.0 L) at 10 °C for 48 h, filtered and then evaporated using a rotary evaporator at below 30 °C. The resultant extract was separated by Diaion® HP-20 (Mitsubishi Chemical Co., Japan). The anthocyanin-rich fraction was freeze-dried (120 g). The supernatant was filtered through a 0.2 mm polytetrafluoroethylene (PTFE) filter, and then subjected to UPLC-QTOF-MS and biological activity analyses. The extraction solvent was of EP grade, whereas the chromatographic solvents used in the MS experiments were of LC–MS grade (J. T. Baker, Phillipsburg, NJ, USA). The purity of PS and PTS was at least 95%.

### 2.2. Regents and Antibodies

Dulbecco’s modified Eagle’s medium (DMEM), fetal bovine serum (FBS) and antibiotics mixture were purchased from WELGENE (Gyeongsan-si, Gyeongsangbuk-do, Korea). Kojic acid (KA), phenylthiourea (PTU), mushroom tyrosinase, 3-(4,5-dimethylthiazol-2-yl)-2,5-diphenyltetrazolium bromide (MTT), α-MSH and PD98059 were purchased from Sigma-Aldrich Co. (St. Louis, MO, USA). Antibodies against tyrosinase, MITF, ERK, phospho-ERK (p-ERK) and anti-β-actin were obtained from Santa Cruz Biotechnology (Santa Cruz, CA, USA). Peroxidase-labeled anti-rabbit and anti-mouse immunoglobulins were obtained from KOMA BIOTECH (Seoul, Korea). All other chemicals were purchased from Sigma-Aldrich. Anthocyanin powder of *H. sabdariffa* L. from Egypt was purchased from Shin Young Hub (Seoul, Korea).

### 2.3. Cell Culture

B16F10 cells (ATCC, Manassas, VA, USA) were maintained in DMEM supplemented with 10% heat inactivated FBS and antibiotics mixture at 37 °C in a humidified atmosphere of 5% CO_2_ and cultured below 15 passage numbers.

### 2.4. Cell Viability

To analyze the effect of PS and PTS on cell viability, the MTT assay was performed. Briefly, B16F10 cells were seeded in 24 well plates at a density of 1 × 10^4^ cell/mL, and then incubated for 18 h at 37 °C. The cells were then treated with different concentrations (0–800 μg/mL) of PS and PTS for 72 h. After incubation, MTT was added to each well and the plates were further incubated for 4 h at 37 °C. The precipitate was dissolved in DMSO and absorbance was measured at 560 nm using a microplate spectrophotometer (Thermo Electron Corp., Marietta, OH, USA).

### 2.5. Flow Cytometry Analysis

To estimate viability, total viable cell count and dead cell percentage, flow cytometry analysis was carried out based on differential staining of viable and non-viable cells due to their different permeability to DNA binding dyes. B16F10 cells were plated at a density of 1 × 10^4^ cell/mL for 18 h and then treated with the indicated concentrations (0–800 μg/mL) of PS and PTS for 72 h. In brief, the cells were harvested and washed with ice-cold phosphate-buffered saline (PBS). Next, the cells were incubated with Muse® cell count and viability kit (EMD Millipore, Billerica, MA, USA) for 5 min and cell viability, total cell count and dead cell population were analyzed by using a Muse® cellcycler (EMD Millipore) according to the manufacturer’s instructions.

### 2.6. Mushroom Tyrosinase Assay

Tyrosinase activity was measured by using mushroom tyrosinase in a cell-free system according to a previous method [24]. Briefly, 130 µL of 100 mM phosphate buffer (pH 6.8), 20 µL of PS or PTS, 30 µL of 1.5 mM L-tyrosine and 20 µL of 210 Units/mL mushroom tyrosinase were mixed. The reaction mixture was then incubated for 30 min at 37 °C, and absorbance was measured at 490 nm by using a microplate spectrophotometer. KA (25 µM) and PTU (250 nM) were used as positive controls.

### 2.7. Measurement of Extracellular and Intracellular Melanin Content

The effect of PS and PTS on α-MSH-induced melanogenesis was measured according to a previous method [25]. Briefly, B16F10 cells were cultured at 1 × 10^4^ cell/mL in a 6 well plate for 18 h and treated with α-MSH (500 ng/mL) for 24 h. Next, the cells were treated with different concentrations of PS and PTS (0–400 μg/mL) for an additional 72 h. After incubation, extracellular melanin content in the culture media was measured. In addition, the cells were washed in ice-cold PBS and dissolved in 1 M NaOH containing 10% DMSO at 100 °C for 10 min, and then absorbance was measured at 405 nm to obtain the melanin content.

### 2.8. Reverse Transcription-Polymerase Chain Reaction (RT-PCR)

B16F10 cells were seeded at 1 × 10^4^ cell/mL in a 6 well plate for 18 h at 37 °C. Next, the cells were pretreated with the α-MSH (500 ng/mL) for 24 h prior to treatment with different concentrations of PS and PTS (0–400 μg/mL). Total RNA was extracted by using an easy-BLUE™ total RNA extraction kit (iNtRON Biotechnology, Seongnam-si, Gyeonggi, Korea) following the manufacturer’s protocol. Two microgram RNA was reverse-transcribed using MMLV reverse transcriptase (Bioneer; Daejeon, Korea) and the target cDNA was amplified using EzWay Neo Taq PCR MasterMix (KOMA BIOTECH). The following primers were used; tyrosinase sense 5′-GTCGTCACCCTGAAAATCCTAACT-3′ and antisense 5′-CATCGCATAAAACCTGATGGC-3′; *MITF* sense 5′-CCCGTCTCTGGAAACTTGATCG-3′ and antisense 5′- CTGTACTCTGAGCAGCAGGTC-3′; glyceraldehyde-3-phosphate dehydrogenase (*GAPDH*) sense 5′-AGGTCGGTGTGAACGGATTTG-3′ and antisense 5′-TGTAGACCATGTAGTTGAGGTCA-3′. The amplification conditions were as follows, for *MITF* and tyrosinase: 95 °C for 30 s, 62 °C for 45 s and extension at 72 °C for 1 min for 25 cycles each; for *GAPDH*: 95 °C for 30 s, 60 °C for 30 s and extension at 72 °C for 30 s. Agarose gel (1.2%) electrophoresis was performed to separate the PCR products and visualized by ethidium bromide (0.01%).

### 2.9. Western Blotting Analysis

B16F10 cells were seeded at 1 × 10^4^ cell/mL in 6 well plates for 18 h at 37 °C and then pretreated with α-MSH (500 ng/mL) for 24 h prior to treatment with different concentrations of PS and PTS (0-400 μg/mL). The cells were lysed with PRO-PREP lysis buffer (iNtRON Biotechnology). The lysate was incubated for 20 min on ice and centrifuged at 16,000 rpm at 4 °C for 15 min. The supernatant was collected and protein concentrations were measured using Bio-Rad protein assay reagents (Bio-Rad, Hercules, CA, USA). Equal amount of protein was separated by electrophoresis on 10% SDS-polyacrylamide gel. The proteins were then transferred to a nitrocellulose membrane (Schleicher and Schuell, Keene, NH, USA) and immunoblotted with specific antibodies overnight at 4 °C. The membrane was washed three times and reincubated with peroxidase-labeled secondary antibody for 2 h. Bound antibodies were detected using an enhanced chemiluminescence plus kit (Thermo Scientific, Rockford, IL, USA). The images were visualized by a Chemi-Smart 2000 (Vilber Lourmat, Marne-la-Vallee, France). Images were captured using Chemi-Capt (Vilber Lourmat) and transported into Adobe Photoshop.

### 2.10. Maintenance and Phenotype Evaluation of Zebrafish

AB strain zebrafish was provided from C.H. Kang (one of the author, Nakdong National Institute of Biological Resources, Sangju, Gyeongsangbukdo, Korea) and cultured at 28.5 °C and a 14/10-h light/dark cycle. To obtain embryos, adult zebrafish (female to male ratio of 1:2) natural spawning was induced in the morning by turning on the light in the spawning room. The collected embryos were placed in embryo medium ((NaCl—34.8 g, KCl—1.6 g, CaCl_2_.2H_2_O—5.8 g and MgCl_2_.6H_2_O—9.78 g) in double-distilled water, pH 7.2) supplemented with 1% methylene blue at 28 °C. Zebrafish aged 2 days post-fertilization (dpf; *n* = 20) was arrayed by using a dropper into 6 well plates containing 2 mL embryo medium. After 2 h of incubation, the culture medium was replaced with new medium containing PS and PTS (400 μg/mL). Spontaneous melanin content was measured by using densitometric analysis of zebrafish larvae at 4 dpf. In a parallel experiment, to investigate the effect of PS and PTS in α-MSH-stimulated zebrafish larvae, the larvae were pretreated with 200 µM PTU for 24 h and then incubated with α-MSH (1 µg/mL) for an additional 24 h. Next, the larvae were treated with different concentrations of PS and PTS at 4 dpf for 48 h. Subsequently, the zebrafish larvae were anesthetized by tricane methane sulfonate solution at 6 dpf, and then mounted in 2% methyl cellulose on a depression slide. Images of the larvae were then captured by using an Olympus SZ2-ILST stereomicroscope (Tokyo, Japan). Densitometric analysis was performed using the Image J software (National Institute of Health). The pigmentation data were expressed as percentage of the value in the untreated control group.

### 2.11. Determination of Cardiotoxicity in Zebrafish

The cardiotoxicity of PS and PTS was determined by comparing the heart rate of zebrafish larvae at 6 dpf, because monitoring zebrafish heart rate is a great tool in drug development and toxicity study [26,27]. Briefly, zebrafish larvae were placed under a stereomicroscope (Olympus SZ2-ILST) for 4 min at room temperature for allowing embryos to acclimate to the light. The heart rate was calculated by counting the number of heart beats in 1 min. The obtained results were expressed as average heart rate per min.

### 2.12. UPLC-QT of MS for Flavonoid Analysis

Chromatographic separation was performed using a UPLC system (Waters Corp., Milford, MA) equipped with a binary solvent delivery system, auto-sampler and UV detector. Aliquots (3.0 μL) of each sample were then injected into a BEH C_18_ column (2.1 × 100 mm, 1.7 μm) at a flow rate of 0.4 mL/min and eluted using a chromatographic gradient of two mobile phases (A: water containing 0.1% formic acid and B: acetonitrile containing 0.1% formic acid). The optimized linear gradient was as follows: 0.0 min, 1% B; 0.0–1.0 min, 1–5% B; 1.0–10.0 min, 5–30% B; 10.0–17.0 min, 30–60% B; 17.0–17.1 min, 60–100% B; 17.1–19.0 min, 100% B and 19.1–20 min, back to 10% B. A quadrupole time-of-flight mass spectrometer (Q-Tof Premier^TM^; Waters Corp., Parsippany, NJ, USA) was operated in the negative ion mode in the following conditions: capillary voltage, 2.3 kV; cone voltage, 50 V; source temperature, 110 °C; desolvation temperature, 350 °C. A lock sprayer was used with the reference solution leucine-enkephalin ([M−H]^−^ *m/z* 554.2615) as the lock mass. The full-scan data and MS/MS spectra were collected by using the MassLynx software (Waters Corp.).

### 2.13. Statistical Analysis

All data represented the mean of at least triplicate experiments, and were expressed as means ± the standard error of the median (SEM). Statistical analysis was performed on the Sigma plot 12.0 software by using the Student’s *t*-test and unpaired one-way analysis of variance (ANOVA) with Bonferroni correction. Statistical significance was set at ^*^ and ^#^ *p* < 0.05, ^**^ *p* < 0.01 and ^***^ and ^###^ *p* < 0.001.

## 3. Results

### 3.1. PS and PTS Do Not Induce Cytotoxicity in B16F10 Cells

**Figure 1:**
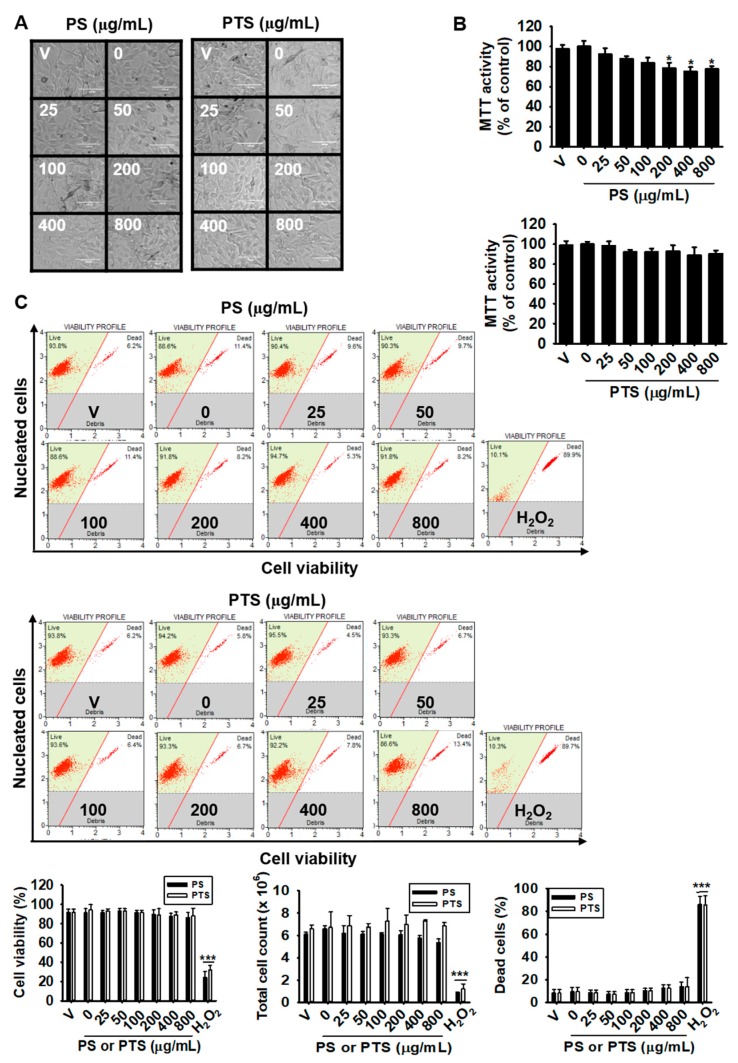
The *Hibiscus syriacus* L. varieties Pulsae and Paektanshim (PS and PTS) do not induce cytotoxicity in B16F10 cells. B16F10 cells were treated with 0–800 µg/mL of PS or PTS for 72 h. (**A**) Microscopic images were captured and analyzed. All scale bars represent 100 μm. (**B**) Mitochondrial activity was determined by the MTT assay. The activity in each group was presented as the percentage values of the untreated control. (**C**) In a parallel experiment, cell viability, total cell count and dead cell population were measured by flow cytometry analysis. H_2_O_2_ (100 μM) was used as a positive control for cell death. Data are reported as the mean ± SEM of three independent experiments (*n* = 3). ^*^ *p* < 0.05 and ^***^ *p* < 0.001, *vs*. untreated group. V; vehicle (0.05% DMSO).

We first investigated whether PS and PTS are cytotoxic. B16F10 cells were treated with various concentrations (0–800 µg/mL) of PS and PTS for 72 h, and cytotoxicity was evaluated by microscopic analysis and an MTT assay. As shown in Figure 1A, no morphological change was observed following treatment with PS and PTS at any concentration, suggesting that PS and PTS did not induce phenotypic change in B16F10 cells. Results of MTT assay showed that PS at the high concentrations (over 200 µg/mL) slightly decreased mitochondrial activity (Figure 1B). To confirm in detail whether PS and PTS influence cell viability and cytotoxicity, flow cytometric analysis was performed (Figure 1C, top). As presented in Figure 1C (middle), PS and PTS, compared with the untreated control, did not increase the population of dead cells, and sustained cell viability and total cell numbers (Figure 1C, bottom). These data indicated that PS and PTS did not exert direct cytotoxicity.

### 3.2. PS and PTS Decrease Extracellular and Intracellular Melanin Production in α-MSH-Stimulated B16F10 Cells

**Figure 2:**
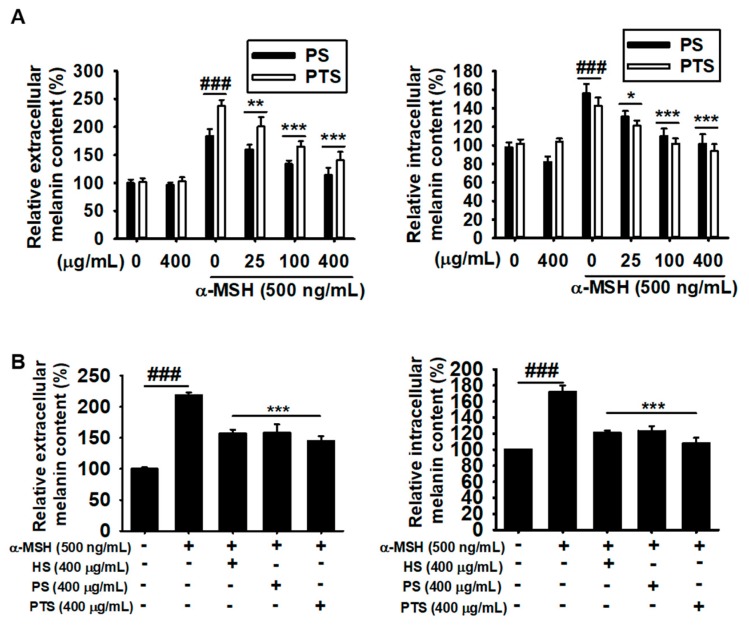
PS and PTS decrease extracellular and intracellular melanin production in α-MSH-stimulated B16F10 cells. (**A**) B16F10 cells were exposed to 500 ng/mL of α-MSH in the presence of 0-400 µg/mL PS or PTS for 72 h, and extracellular (right) and intracellular (left) melanin contents were measured. (**B**) In a parallel experiment, the cells were treated with α-MSH (500 ng/mL), PS (400 µg/mL), PTS (400 µg/mL) or commercial *H. sabdariffa* L. anthocyanin (HS, 400 µg/mL) for 72 h, and extracellular (right) and intracellular (left) melanin contents were measured. The percentage values in each group are relative to those in the untreated control. Data are reported as the mean ± SEM of three independent experiments performed (*n* = 3). ^###^ *p* < 0.001 *vs*. untreated group; ^*^ *p* < 0.05, ^**^ *p* < 0.01 and ^***^ *p* < 0.001 *vs*. α-MSH-stimulated group.

To investigate the effect of PS and PTS on melanogenesis, B16F10 cells were treated with various concentrations (0–400 µg/mL) of PS and PTS for 72 h, and melanin content was measured from extracellular and intracellular compartments. Under no stimulation of α-MSH, melanin contents in both compartments in the groups treated with PS and PTS at 400 µg/mL were comparable to those in the untreated group (Figure 2A). Treatment with α-MSH significantly increased extracellular and intracellular melanin content to approximately 200% (Figure 2A, left) and 150% (Figure 2A, right) compared with those of the untreated group. However, PS and PTS downregulated the α-MSH-mediated increases in extracellular and intracellular melanin content in a dose-dependent manner. Next, the effectiveness of commercial *H. sabdariffa* L. anthocyanins (HS) in inhibiting melanogenesis was compared to that of PS and PTS. As shown in Figure 2B, we found that both PS and PTS remarkably decreased the extracellular and intracellular melanin comparable to those of HS-treated group. These data indicated that PS and PTS would be promising candidates of an anti-melanogenic agent.

### 3.3. PS and PTS Do Not Downregulate Mushroom Tyrosinase Activity in Vitro

**Figure 3:**
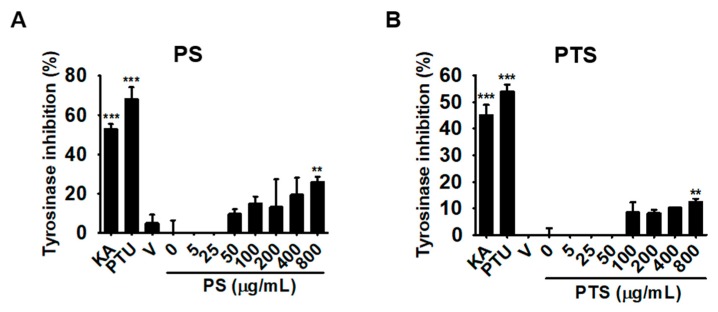
PS and PTS do not downregulate mushroom tyrosinase activity *in vitro*. The inhibitory activity of PS (**A**) and PTS (**B**) on mushroom tyrosinase was measured *in vitro*. PS or PTS (0–800 µg/mL), kojic acid (KA, 25 µM) and PTU (250 nM) were loaded onto a 96-well microplate. After incubation with mushroom tyrosinase at 37 °C for 30 min, dopaquinone level was measured by spectrophotometry at 490 nm. The percentage values in each experiment are expressed relative to those of untreated control. Data are reported as the mean ± SEM of three independent experiments performed (*n* = 3). ^**^ *p* < 0.01 and ^***^ *p* < 0.001 *vs.* untreated group. V, vehicle (0.01% DMSO).

Mushroom tyrosinase is a widely used tool for determining the effect of potential melanogenesis inhibitors as candidate treatments of some dermatological disorders and as skin-whitening agents. Therefore, we investigated whether PS and PTS negatively regulate mushroom tyrosinase activity *in vitro* through *O*-hydroxylation of L-tyrosine and/or through oxidation of L-DOPA to *O*-diquinone. Well-known tyrosinase inhibitors, KA and PTU, significantly inhibited mushroom tyrosinase activity to approximately 50% and 60%, respectively; however, both of PS and PTS did not inhibit the activity, and even at the highest concentration (800 μg/mL), they only slightly increased tyrosinase inhibitory activity (Figure 3A,B). These data indicated that low concentrations of PS and PTS did not directly inhibit tyrosinase activity.

### 3.4. PS and PTS Inhibit the Expression of MITF and Tyrosinase in α-MSH-Stimulated B16F10 Cells

**Figure 4:**
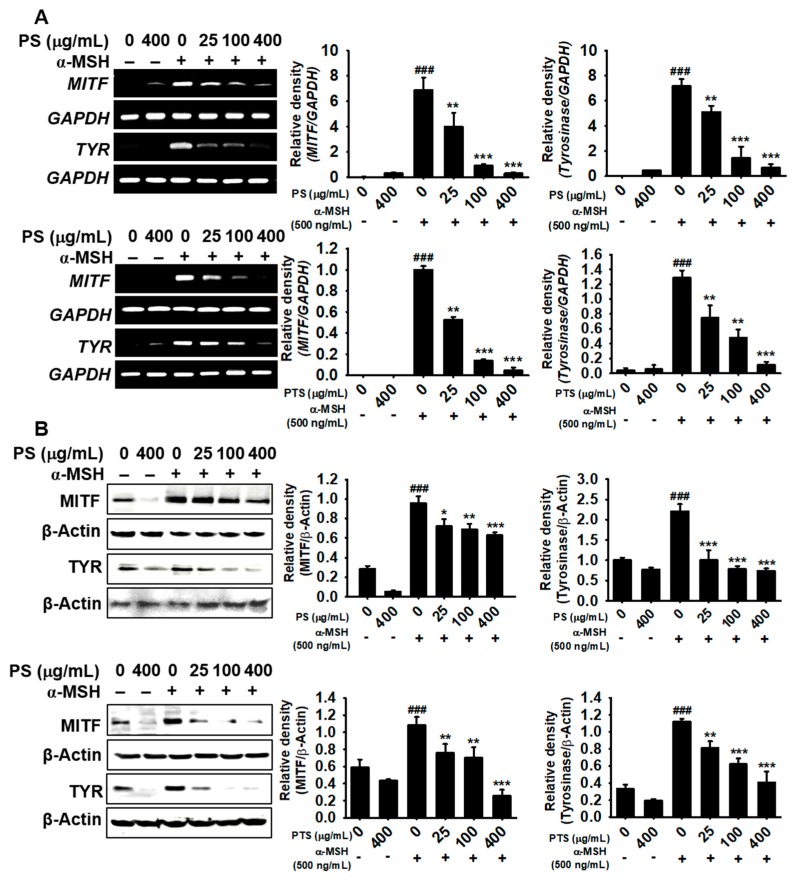
PS and PTS inhibit the expression of MITF and tyrosinase in α-MSH-stimulated B16F10 cells. (**A**) B16F10 cells were exposed to 500 ng/mL of α-MSH in the presence of PS or PTS (0–400 µg/mL) for 48 h, and the gene expression of *MITF* and tyrosinase (*TYR*) was measured. (**B**) Under the same experimental condition, the protein expression of MITF and TYR was measured by western blotting analysis at 72 h. The data are relative to the values in the untreated control group and represented as the means ± SEM of three separate experiments (*n* = 3). ^###^ *p*< 0.001 *vs.* untreated group; ^*^ *p* < 0.05, ^**^ *p* < 0.01 and ^***^ *p* < 0.001 *vs.* α-MSH-stimulated group.

To investigate whether PS and PTS affect the expression of key regulators in melanogenesis such as MITF and tyrosinase, RT-PCR and western blotting analysis were performed after treatment with PS and PTS. As shown in Figure 4A, α-MSH significantly upregulated *MITF* and tyrosinase expression at 48 h and both of PS and PTS suppressed α-MSH-induced *MITF* and tyrosinase expression in a dose-dependent manner. Especially, PS and PTS at the highest concentration (400 μg/mL) substantially reduced α-MSH-induced *MITF* and tyrosinase expression to the level observed in the untreated group. In addition, both PS and PTS also decreased the α-MSH-induced increase in the protein levels of MITF and tyrosinase at 72 h (Figure 4B). These results suggested that PS and PTS inhibited α-MSH-mediated melanogenesis by suppressing the expression of MITF and tyrosinase.

### 3.5. PS and PTS Inhibit Melanin Synthesis in Zebrafish Larvae

**Figure 5:**
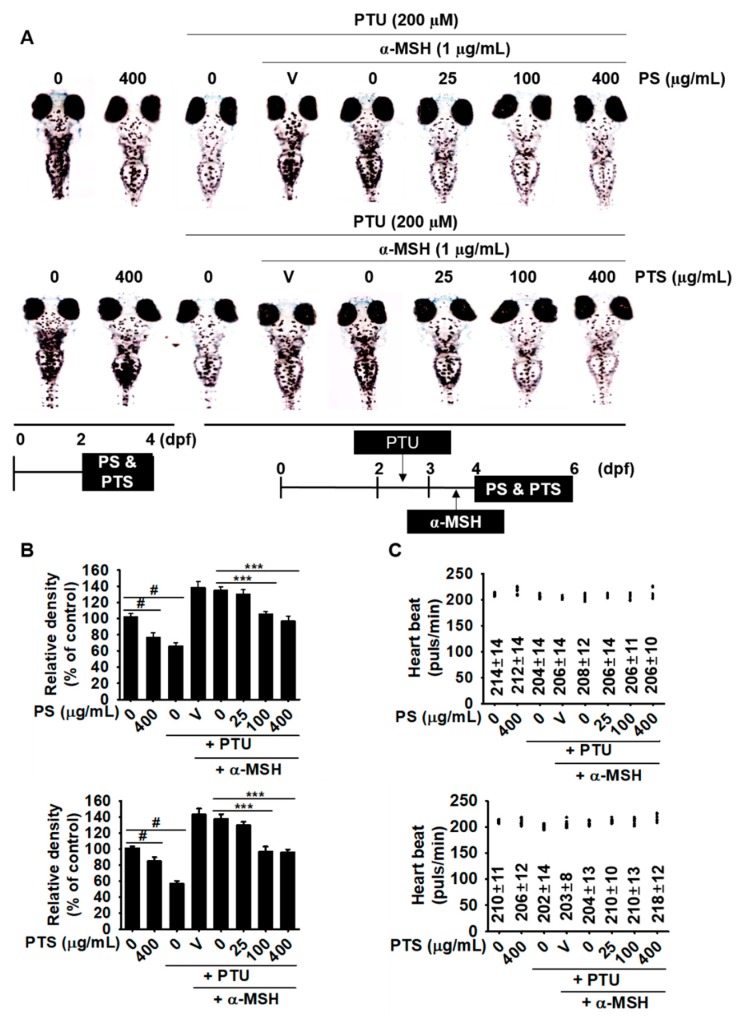
PS and PTS inhibit melanin synthesis in zebrafish larvae. (**A** and **B**) Zebrafish larvae at 2 dpf were treated with PS and PTS (400 µg/mL) for 48 h, and images were collected (left two zebrafish larvae, top for PS and bottom for PTS). Additionally, 2 dpf zebrafish larvae (*n* = 20) were treated with PTU (200 µM) for 24 h, and the medium was replaced with α-MSH (1 µg/mL) for another 24 h. Next, the larvae were treated with the indicated concentrations of PS or PTS for 48 h. The effects of PS and PTS on pigmentation in zebrafish larvae were observed under an Olympus microscope (40×). (**B**) Relative density was calculated by the Image J software. (**C**) Average heart rate of zebrafish larvae (*n* = 20) was measured to assess the toxicity of PS and PTS. Data are reported as the mean ± SEM. ^#^ *p* < 0.05 *vs.* untreated group; ^***^ *p* < 0.001 *vs.* α-MSH-stimulated group. V, vehicle control (0.05% DMSO).

To further evaluate the anti-melanogenic activity of PS and PTS, we used α-MSH-stimulated zebrafish larvae in the presence or absence of PS and PTS. First, 2-dpf zebrafish larvae were treated with PS and PTS for 2 days, and the results showed the anti-melanogenic activity of PS and PTS (Figure 5A, left two zebrafish larvae of top and bottom). In addition, we investigated whether PS and PTS downregulate α-MSH-stimulated melanogenesis in zebrafish larvae. Zebrafish larvae at 2 dpf were pretreated with PTU (200 µM) for 24 h to reduce background pigmentation and then treated with α-MSH (1 µg/mL) for an additional 24 h. At 4 dpf, the larvae were treated with PS and PTS for 48 h. As expected, both PS and PTS significantly decreased melanin pigmentation in a dose-dependent manner (Figure 5A); both PS and PTS at 400 µg/mL significantly reduced melanin pigmentation to approximately 40%, compared to that in α-MSH-stimulated group (Figure 5B). To determine whether PS and PTS exert cardiotoxicity in zebrafish larvae, we monitored heart rate, morphological patterns and mortality. The zebrafish larvae treated with PS (Figure 5C, top) and PTS (Figure 5C, bottom) did not show any obvious difference in heart rate compared to the untreated control group. Furthermore, no morphological malformation and mortality of the larvae were observed after treatment with PS and PTS for 48 h (Figure 5A). These results suggested that PS and PTS were potent inhibitors of melanogenesis *in vivo*.

### 3.6. PS- and PTS-induced ERK Phosphorylation Downregulates MITF Expression

**Figure 6:**
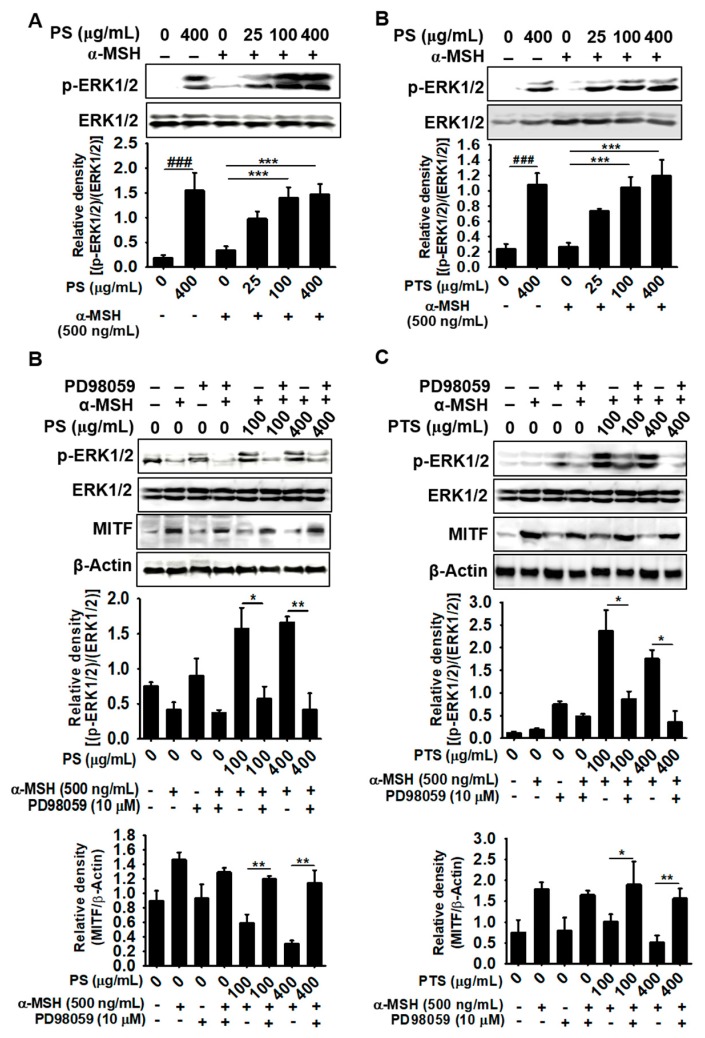
PS- and PTS-induced ERK phosphorylation downregulates MITF expression. (**A**,**B**) B16F10 cells were pretreated with 500 ng/mL of α-MSH and then treated with PS or PTS (each 0–400 µg/mL) for 72 h, and ERK phosphorylation was analyzed by western blotting analysis (A, PS-treated; B, PTS-treated). (**C**,**D**) The cells were pretreated with PD98059 (10 μM) for 1 h, and then treated with 500 ng/mL of α-MSH in the absence or presence of PS or PTS (each 100 μg/mL and 400 μg/mL) for 72 h. Next, the levels of p-ERK1/2 and MITF in cell lysate were analyzed by western blotting analysis (C, PS-treated; D, PTS-treated). The percentage values are relative to those in the untreated group. The data are represented as the means ± SEM of three separate experiments (*n* = 3). ^###^ *p* <0.001 *vs.* untreated group; ^*^ *p* < 0.05, ^**^ *p* < 0.01 and ^**^ *p* < 0.001 *vs.* α-MSH-stimulated group.

Since ERK phosphorylation inhibits tyrosinase activity by enhancing the proteasomal degradation of MITF and leads to inhibition of melanogenesis [13,28], we examined whether PS and PTS induce ERK phosphorylation in α-MSH-stimulated B16F10 cells. Our results revealed that no significant expression of ERK phosphorylation occurred in α-MSH-stimulated B16F10 cells; however, PS (Figure 6A) and PTS (Figure 6B) remarkably upregulated ERK phosphorylation, irrespective of the presence of α-MSH. Next, we examined whether a specific ERK inhibitor, PD98059, restores MITF expression. Additionally, both PS (Figure 6C) and PTS (Figure 6D) diminished MITF expression and significantly increased ERK phosphorylation; however, PD98059 enhanced MITF expression in both PS- and PTS-treated B16F10 cells and downregulated ERK phosphorylation. Therefore, these results indicated that ERK phosphorylation was involved in the downregulation or degradation of MITF by PS and PTS in B16F10 cells.

### 3.7. The ERK Signaling Pathway Downregulates Melanogenesis in PS- and PTS-treated B16F10 Cells and Zebrafish Larvae

**Figure 7:**
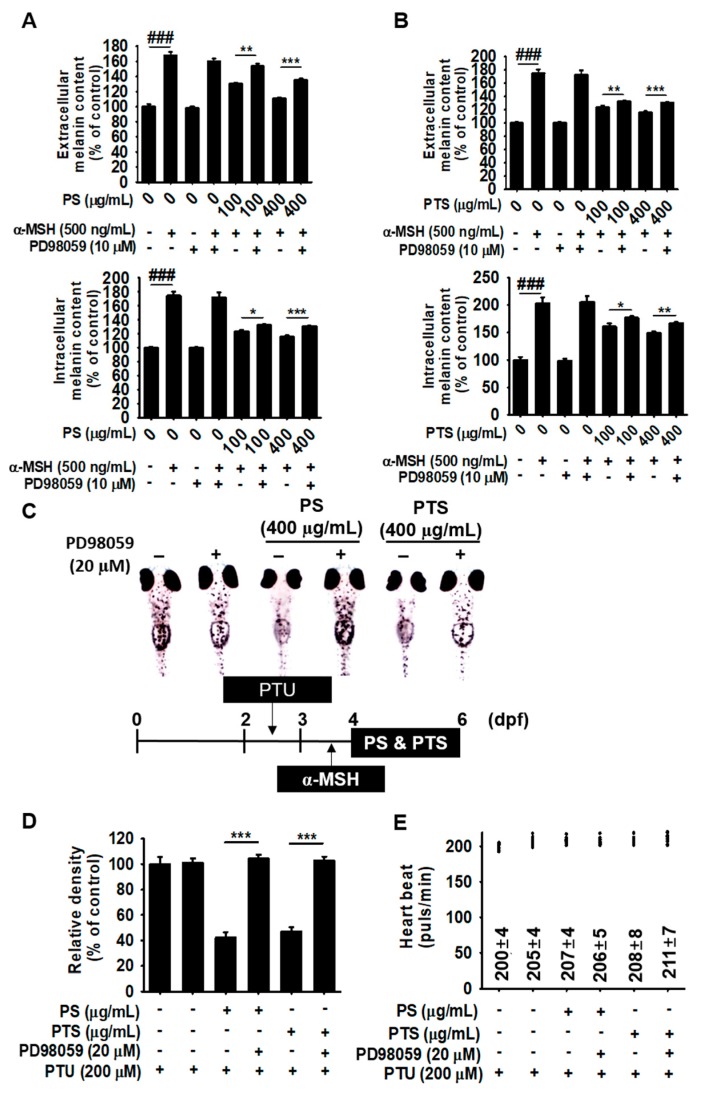
The ERK signaling pathway inhibits melanogenesis in PS- and PTS-treated B16F10 cells and zebrafish larvae. (**A**,**B**) B16F10 cells were treated with α-MSH (500 ng/mL) in the presence of PS or PTS (100 µg/mL and 400 µg/mL) for 72 h after pretreatment with PD98059 (10 µM). Extracellular (top) and intracellular (bottom) melanin contents were measured (A: PS-treated; B; PTS-treated). (**C**) Zebrafish at 2 dpf (*n* = 20) were treated with PTU (200 μM) for 24 h and then with α-MSH (1 µg/mL) for 48 h. Next, the medium was replaced with PD98059 (20 μM) for 2 h, and then the fish were treated with PS (400 µg/mL) or PTS (400 µg/mL) for 48 h. (**D**) Pigmentation in zebrafish was observed under an Olympus microscope (40×) and relative density was calculated by the Image J software. (**E**) Average heart rate in zebrafish larvae (*n* = 20) was measured to assess the toxicity of the extracts. Data are reported as the mean ± SEM of three independent experiments (*n* = 3). ^###^ *p* < 0.001 *vs*. untreated group; ^*^ *p* < 0.05, ^**^ *p* < 0.01 and ^***^ *p* < 0.001 *vs*. α-MSH-stimulated group (A and B) and *vs*. PTU + PT or PTS group (D).

To further confirm the role of the ERK signaling pathway in the anti-melanogenic effects of PS and PTS, we examined extracellular and intracellular melanin content in α-MSH-stimulated B16F10 cells and melanogenesis in zebrafish larvae. As shown in Figure 7A,B, PD98059 did not regulate α-MSH-mediated increase in extracellular and intracellular melanin content, which indicated that PD98059 did not influence α-MSH-stimulated melanin production because the ERK signaling pathway deviates from canonical α-MSH-stimulated melanogenesis. On the contrary, PD98059 directly reversed PS- and PTS-mediated inhibition (Figure 7A,B, respectively) in extracellular (top) and intracellular (bottom) melanin contents. These results implied that PS and PTS inhibited melanin production in α-MSH-stimulated B16F10 cells through the ERK signaling pathway. Furthermore, to evaluate whether the anti-melanogenic activity of PS and PTS occurred by activating ERK, zebrafish larvae were treated with PS, PTS or PD98059 for 48 h after pretreatment with α-MSH for 24 h. As shown in Figure 5A, PS and PTS remarkably suppressed α-MSH-stimulated melanogenesis in zebrafish larvae; however, PD98059 significantly increased melanin pigmentation on the body surface of PS- and PTS-treated zebrafish larvae (Figure 7C). Compared with the PS- and PTS-treated groups (each at 400 µg/mL), PD98059 treatment increased melanogenesis to approximately 124% and 111%, respectively (Figure 7D). PS and PTS showed no significant effect on heart rate in zebrafish and did not exhibit any conspicuous adverse effect (Figure 7E). Moreover, the heart rate was regularly sustained, suggesting that all chemicals did not exert toxicity under the current experimental conditions (Figure 7E). These data indicated that ERK activation was a negative key regulator of the anti-melanogenic effect of PS and PTS.

### 3.8. Tentative Identification of Metabolites in PS and PTS

To determine the importance of analyzed metabolites in a practical content, the anthocyanin and flavonoid profiles of PS and PTS were analyzed by UPLC-PDA-QTof-MS, and the results are shown in Figure S1 (top, PS; bottom, PTS) and Table 1. The typical fragmentation pattern included *O*-glucoside (m/z 162), C-glucoside (m/z 120 and 90) and acetylhexoside (m/z 204) [29]. The peaks 1, 2 and 4 were tentatively identified as cyanidin-3-*O*-galatoside (1, tR = 4.34 min, m/z 449, 287), cyanidin-3-*O*-glucoside (2, tR = 4.43 min, m/z 449, 287) and cyanidin-3,5-*O*-diglucoside (4, tR = 4.87 min, m/z 611, 449 and 287), respectively, because of their short retention times and according to comparison with MS/MS data in the literature [30,31]. The fragment ion of peaks 3 and 5–12 showed the typical characteristics of C-glycosyl flavones, such as loss of −90 and −120 amu. In addition, the product ion mass spectra of peaks 3 (tR = 4.61 min, m/z 609), 5 (tR = 5.21 min, m/z 609), 6 (tR = 5.32 min, m/z 593), 7 (tR = 5.43 min, m/z 739), 8 (tR = 5.54 min, m/z 593), 9 (tR = 6.02 min, m/z 431), 10 (tR = 6.20 min, m/z 577), 11 (tR = 6.28 min, m/z 431) and 12 (tR = 6.50 min, m/z 635) were shown as deprotonated ion [M–H]^−^ and the main fragment ion at m/z 447 [(M–H)–162]^−^, 431 [(M–H)–162]^−^, m/z m/z 357 [(M–H)–(162+90)]^−^ and m/z 327 [(M–H)–(162+120)]^−^. This molecular weight was determined to be the characteristic of apigenin in the aglycone moiety, according to comparison with previously published data [31]. Therefore, peaks 3 and 5–12 were confirmed as orientin-7-*O*-glucoside (3), isoorientin-4′-*O*-glucoside (5), isovitexin-4′-*O*-glucoside (6), vitexin-4′-*O*-glucoside-2″-*O*-rhamnoside (7), isovitexin-7-*O*-glucoside (8), apigenin-8-C-β-D-glucoside (9), isovitexin-2″-*O*-rhamnoside (10), apigenin-6-C-β-D-glucoside (11) and apigenin-6-C-glucoside-7-(6″-*O*-acetylglucoside) (12). Peak 6 was present in very high intensity because it appeared as a principal peak in the UPLC-PDA-QTof-MS chromatogram with retention times. Based on MS/MS analysis, the spectra of peaks 13–17 contained ions at m/z 284–285 and at 207–271, indicating that their aglycone moieties were kaempferol and apigenin. Peak 13 (tR = 6.94 min, m/z 693) had a molecular ion [M−H]^−^ at m/z 693 and produced MS/MS fragments at m/z 447, 284 and 255, a typical fragment of kaempferol-*O*-glucoside. Based on these results, compound 13 was identified as a kaempferol-*O*-glucoside derivative. Peaks 14–17 were identified as kaempferol-7-*O*-glucoside (14; tR = 7.23 min, m/z 447, 284), kaempferol-3-*O*-glucoside (15; tR = 7.45 min, m/z 447, 285), apigenin-7-*O*-glucoside (16; tR = 7.53 min, m/z 431, 271) and kaempferol-3-(6″-*O*-acetylglucoside) (17; tR = 4.43 min, m/z 489, 284), respectively, by matching the experimental MS, MS/MS and molecular data with those in the literature [32]. These glycones were previously reported as common flavonoid glycosides. Their structures are shown in Figure S2.

## 4. Discussion

**Figure 8:**
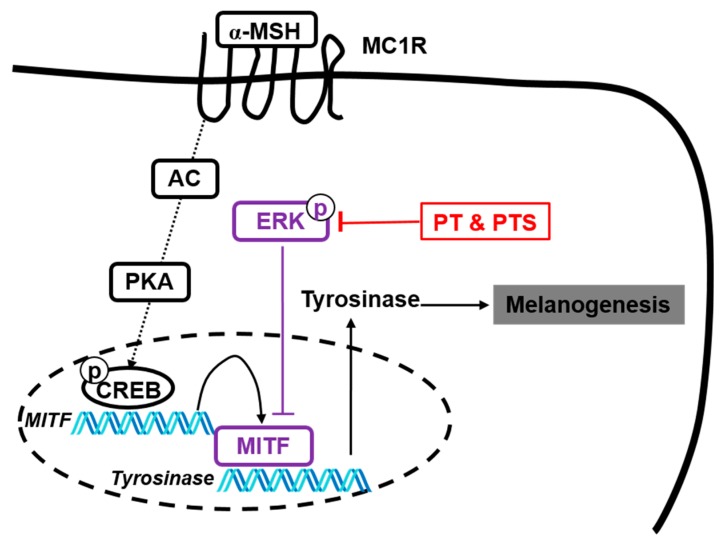
The anti-melanogenic mechanism of PS and PTS. PS and PTS inhibits melanin production in B16F10 cells and zebrafish larvae by activating the ERK signaling pathway, which consequently downregulates tyrosinase expression and activity by inhibiting MITF expression. AC, adenylyl cyclase; PKA, protein kinase A.

Anthocyanins are color pigments in most fruits, vegetables and flowers and possess beneficial effects against many chronic diseases, such as diabetes, cardiovascular disease and obesity [29,33]. Over the past decades, many researchers have investigated the potential health benefits of anthocyanins [34,35]. Recently, purified anthocyanins from *H. sabdariffa* showed to exert antioxidant and antiproliferative activities [36,37], and reduced low-density lipoprotein-mediated macrophage apoptosis and hepatic damages [38,39,40]. Nevertheless, there has been no report on the effects of anthocyanins from *H. syriacus* L. *H. syriacus* L. (rose of Sharon or Mugunghwa), a well-known traditional medicine and a member of the Malvaceae family with *H. sabdariffa* L., has numerous pharmacological benefits. In the current study, for the first time, we confirmed that anthocyanin of two *H. syriacus* L. varieties (PS and PTS, respectively) contained 17 anthocyanins that were different from those isolated from *H. sabdariffa* L. and revealed anti-melanogenic activity by activating the ERK signaling pathway (Figure 8). The anti-melanogenic activity of PS and PTS at 400 μg/mL was also similar level to that of arbutin (1 mM) in B16F10 cells and the zebrafish larvae (Figure S3), which indicating that PS and PTS are promising anti-melanogenic agents.

Tyrosinase is a multifunctional copper-containing enzyme that positively regulates melanin production. Therefore, during several decades, many natural tyrosinase inhibitors with potentials for cosmetic and medical applications have been found. Especially, monophenolic compounds, including hydroquinone, arbutin, resorcinol and KA have been known to directly bind to tyrosinase instead of its substrates, such as L-tyrosine and DOPA, which have a monophenolic structure [41]. Furthermore, many polyphenols exert potent anti-melanogenic compounds by directly binding to tyrosinase via hydrogen bonds and van der Waals forces, as well as owing to their structural specificity, such as the position of their hydroxyl groups [42]. Among polyphenols, anthocyanin from black soya bean showed inhibitory effect on the activities of human and mushroom tyrosinase [43]. The soy bean anthocyanins contained delphinidin-3-glucoside (9.1%), cyanidin-3-*O*-glucoside (76.6%) and peonidin-3-glucoside (9.3%), which significantly downregulated tyrosinase activity *in vitro*; PS and PTS possesses 17 different anthocyanins, which is approximately 5% cyaniding-3-*O*-gucoside and void of delphinidin-3-glucoside and peonidin-3-glucoside. In the current study, we purified anthocyanin-rich extracts of two *H. syriacus* varieties and confirmed that the extracts at a high concentration of 800 μg/mL moderately inhibited mushroom tyrosinase activity and molecular docking analysis showed that PS and PTS did not directly bind to mushroom tyrosinase (Figure S4). In addition, both PS and PTS significantly suppressed extracellular and intracellular melanin contents in B16F10 cells and melanin production in zebrafish larvae, suggesting that both PS and PTS negatively regulated melanin production through alternative mechanisms, not through direct inhibition of tyrosinase activity. This discrepancy implies that some anthocyanins directly targets tyrosinase substrates such as L-tyrosine and DOPA, not tyrosinase. In addition, the heart rate in zebrafish larvae is a promising substitute model for evaluating drug-induced potential cardiotoxicity [26], which has been reliable to identify toxicity to the human system [27]. Therefore, the heart rate was evaluated whether PS and PTS exert cardiotoxicity in zebrafish larvae. No considerable difference of the heart rate was observed in PS- and PTS-treated zebrafish larvae compared with that of the untreated group, which indicates that PS and PTS do not exhibit any significant hostile effect in zebrafish larvae.

Recently, the ERK pathway was found to directly stimulate MITF phosphorylation, causing MITF proteasomal degradation, which consequently inhibits melanogenesis [6,28]. Therefore, ERK activation has been thought as a promising target in anti-melanogenesis treatment. In the current study, we found that PS and PTS significantly activated ERK phosphorylation, and that PD98059, an ERK inhibitor, reverted the anti-melanogenic effects of PS and PTS in B16F10 cells and zebrafish larvae, as well as increased MITF level, thereby indicating that the anti-melanogenic activity of PS and PTS was due to increased ERK phosphorylation and subsequent degradation of MITF. Nevertheless, we could not find the target molecule of PS and PTS in ERK phosphorylation during the anti-melanogenic effect. Therefore, we tried to find negative regulators of the ERK signaling pathway that can bind to the anthocyanins of PS and PTS, thus causing ERK activation. We used the first well-known negative regulator of the ERK pathway, namely a protein phosphatase 2A (PP2A) [44]. Molecular docking study found that PP2A (PDB: 3FGA) did not directly bind to all the anthocyanins identified from PS and PTS, suggesting that PP2A is not a target molecule of PS and PTS. Additionally, Buffet *et al.*, reported two ERK-specific dual specificity phosphatases (DUSP5 and DUSP6) induce dephosphorylation of ERK in the nucleus and cytoplasm [45]. However, all anthocyanins did not bind to DUSP5 (PDB: 2G6Z) and DUSP6 (PDB: 1HZM). Next, DUSP7 (PDB: 4Y2E) is also a well-known ERK-specific phosphatase that can dephosphorylate ERK [46,47]. Even though molecular docking results showed four different poses for the binding of each anthocyanin and DUSP7, it was difficult to pinpoint which amino acids of DUSP7 could accurately bind to the anthocyanins identified in this study because of more than two coplanar positions of DUSP7. Further study will be needed to find accurate amino acids of DUSP7 binding with PS and PTS. Nevertheless, based on the docking score (Table S1), apigenin-7-*O*-glucoside (docking score: −7.4), vitexin-4′-*O*-glucoside-2″-*O*-rhamnoside (−7.3), kaempferol-7-*O*-glucoside (−7.3) and apigenin-6-C-glucoside-7-(6″-*O*-acetyl)-glucoside (−7.2) had strong binding activity to DUSP7, whereas kaempferol-3-*O*-glucoside (−5.8), orientin-7-*O*-glucoside (−5.7) and isovitexin-7-*O*-glucoside (−5.5) had relatively low binding activity. These data suggested that anthocyanins from PS and PTS promoted anti-melanogenic activity by activating the ERK signaling pathway. Previous research also showed that the inhibition of the AC and PKA pathway positively modulated ERK activation [48], thus suggesting that AC inhibition would be one of the upstream targets of anthocyanins from PS and PTS, leading to ERK-mediated anti-melanogenesis effect. Nevertheless, some questions in this study remained to be solved. As mentioned above, many evidence showed that ERK activators are promising candidates of anti-melanogenesis agents [6,28], and our data verified that anthocyanins from PS and PTS inhibited melanogenesis by phosphorylating ERK, irrespective of α-MSH stimuli. However, in other experimental conditions, some anthocyanins suppressed the ERK pathway, leading to anti-cancer and anti-metastasis activities [49,50]. Thus, it is still unclear whether anthocyanins increase or decrease ERK phosphorylation in different experimental conditions. Further studies on anthocyanins in different experimental models are therefore required.

## 5. Conclusions

PS and PTS possessed 17 anthocyanins, which led to ERK phosphorylation and, ultimately, melanogenesis inhibition. Thus, PS and PTS would be potential skin-whitening agents and novel therapeutic agents for treating dermatological problems such as melasma, wrinkling and senile lentigines.

## Figures and Tables

**Table 1:** Molecular formula, retention time, calculated ion, fragments and PubChem compound ID (CID) from PS and PTS.

NO.	I.D.	Molecular Formula	Retention Time (min)	Calculated Ion (*m*/*z*)	Fragments	PubChem CID
1	Cyanidin-3-*O*-galactoside	C_12_H_21_O_11_^+^	4.34	449.1084	259, 287, 421	441699
2	Cyanidin-3-*O*-glucoside	C_21_H_21_O_11_^+^	4.43	449.1084	259,287. 421	44256715
3	Orientin-7-*O*-glucoside	C_27_H_30_O_16_	4.61	609.1456	327, 357, 447	44257973
4	Cyanidin-3,5-*O*-diglucoside	C_27_H_31_O_16_^+^	4.87	611.1612	259, 287, 449	44256718
5	Isoorientinm-4′-*O*-glucoside	C_27_H_30_O_16_	5.21	609.1456	193, 285, 299, 327, 357, 447	44257975
6	Isovitexin-4′-*O*-glucoside	C_27_H_30_O_15_	5.32	593.1506	116, 447	154105
7	Vitexin-4′-*O*-glucoside-2″-*O*-rhamnoside	C_33_H_40_O_19_	5.43	739.2086	431, 447, 593	44257755
8	Isovitexin-7-*O*-glucoside (saponarin)	C_27_H_30_O_15_	5.54	593.1506	283, 311, 431	441381
9	Apigenin-8-C-β-D-glucopyranoside (Vitexin)	C_21_H_20_O_10_	6.02	431.0987	283, 311	5280441
10	Isovitexin-2″-*O*-rhamnoside	C_27_H_30_O_14_	6.20	577.1557	293, 311, 431	44257672
11	Apigenin-6-C-β-D-glucopyranoside (Isovitexin)	C_21_H_20_O_10_	6.28	431.0978	283, 311, 341	162350
12	Apigenin-6-C-glucoside-7-(6″-*O*-acetyl)-glucoside	C_29_H_32_O_16_	6.50	635.1671	431	44257840
13	Kaempferol-*O*-glucoside derivative	C_31_H_34_O_18_	6.94	693.1612	227, 255, 284, 300, 311	*N.F.
14	Kaempferol-7-*O*-glucoside	C_21_H_20_O_11_	7.23	447.0927	227, 255, 285	10095180
15	Kaempferol-3-*O*-glucoside	C_21_H_20_O_11_	7.45	447.0927	151, 257, 285	44258798
16	Apigenin-7-*O*-glucoside	C_21_H_20_O_11_	7.53	431.0978	268, 271	44257792
17	Kaempferol-3-(6″-acetylglucoside)	C_23_H_22_O_12_	7.85	489.1033	227,255, 284, 429	44258855

*N.F.; not found.

## Supplementary Materials

The supplementary materials are available online at https://www.mdpi.com/2218-273X/9/11/645/s1.
