# Supplementary material for: Anthocyanins from Hibiscus syriacus L. Inhibit Melanogenesis by Activating the ERK Signaling Pathway
Source: Biomolecules. 2019 Oct 24;9(11):645. doi: 10.3390/biom9110645 (PMC6920888; doi:10.3390/biom9110645)
Supplement: Supplementary file 1 [file biomolecules-09-00645-s001.pdf]

biomolecules

MDPI

Supplementary Materials: Anthocyanins from *Hibiscus syriacus* L. Inhibit Melanogenesis by Activating the ERK Signaling Pathway

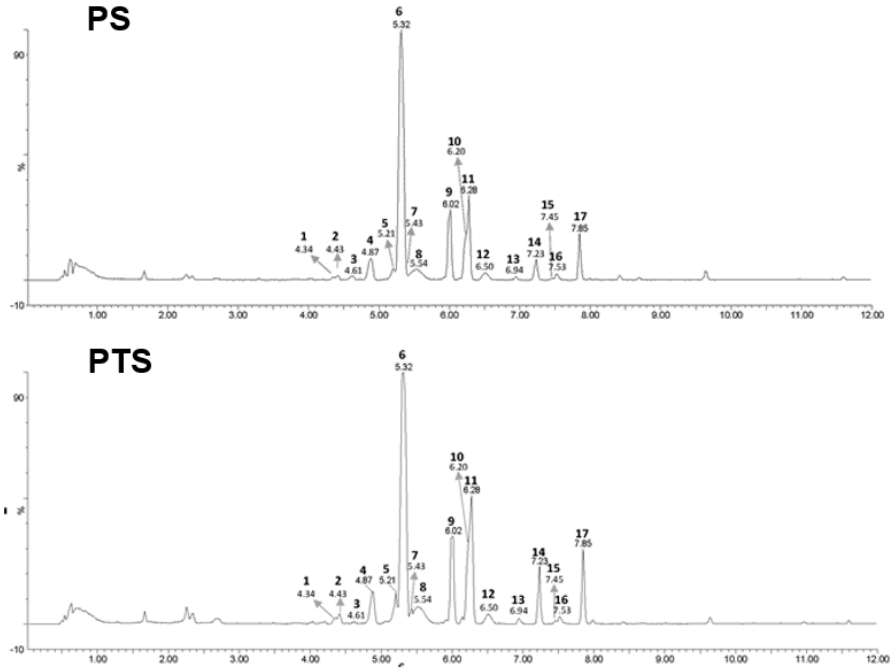

Figure 1. Comprehensive profile of anthocyanin and flavonoid constituents in PS and PTS was directly analyzed by UPLC-PDA-QTof-MS chromatogram.

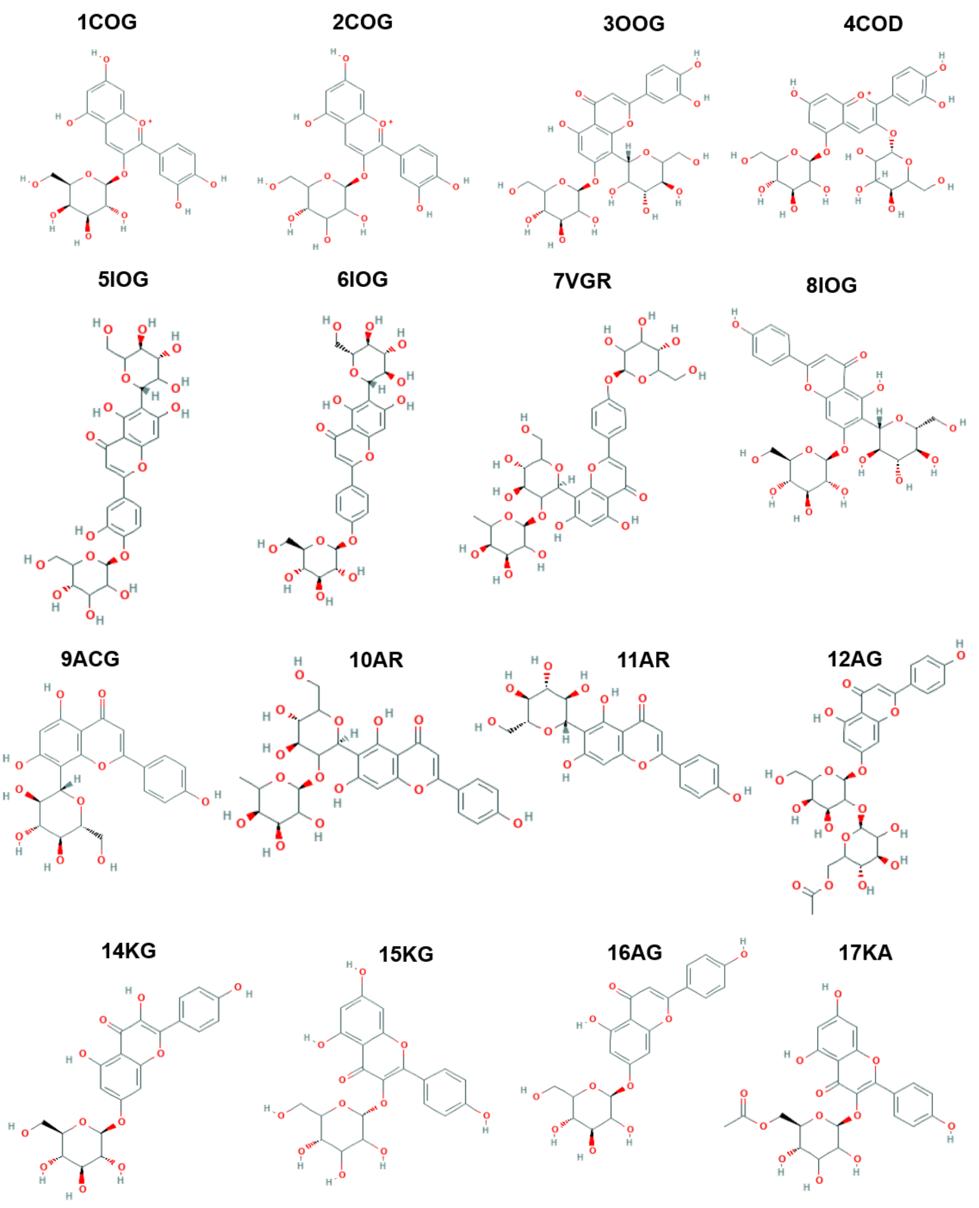

Figure S2. Structure of 17 flavonoid glycosides confirmed from *H. syriacus* in this study. 1COG, Cyanidin-3-*O*-galactoside; 2COG, Cyanidin-3-*O*-glucoside; 3OOG, Orientin-7-*O*-glucoside; 4COD, Cyanidin-3,5-*O*-diglucoside; 5IOG, Isoorientinm-4'-*O*-glucoside; 6IOG, Isovitexin-4'-*O*-glucoside; 7VGR, Vitexin-4'-*O*-glucoside-2''-*O*-rhamnoside; 8IOG, Isovitexin-7-*O*-glucoside (saponarin); 9ACG, Apigenin-8-C-β-D-glucopyranoside (Vitexin); 10IR, Isovitexin-2''-*O*-rhamnoside; 11AG, Apigenin-6-C-β-D-glucopyranoside (Isovitexin); 12AG, Apigenin-6-C-glucoside-7-(6''-*O*-acetyl)-glucoside; 13KG, Kaempferol-*O*-glucoside derivative (not found in PubChem); 14KG, Kaempferol-7-*O*-glucoside; 15KG, Kaempferol-3-*O*-glucoside; 16AG, Apigenin-7-*O*-glucoside; 17KA, Kaempferol-3-(6''-acetylglucoside).

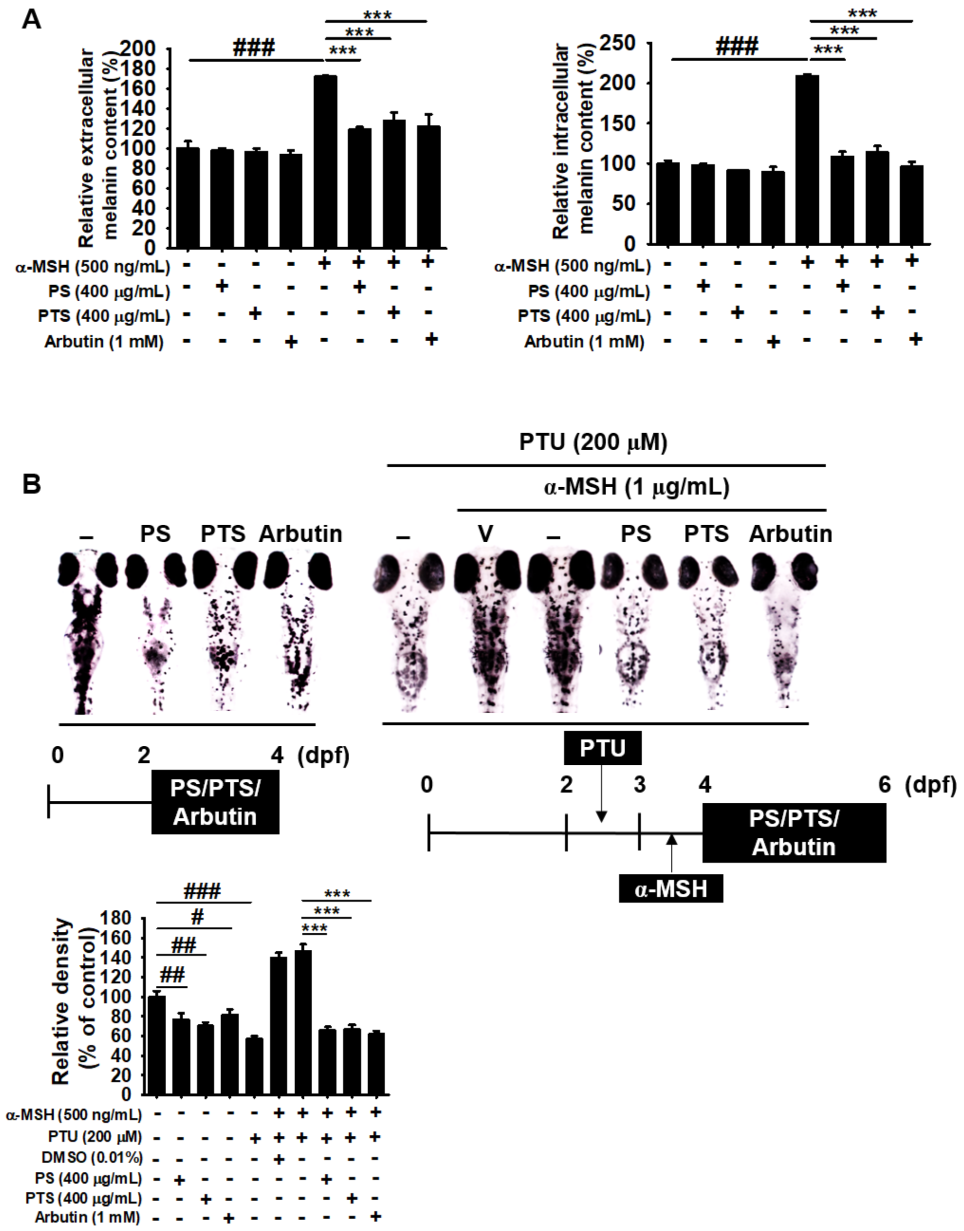

Figure S3. PS and PTS decrease melanogenesis in α-MSH-stimulated B16F10 cells and inhibit the melanin synthesis in zebrafish larvae. (A) B16F10 cells were exposed to 500 ng/mL of α-MSH in the presence of PS or PTS (400 μg/mL) and 1 mM arbutin for 72 h, and extracellular (right panel) and intracellular (left panel) melanin contents were measured. (B). Zebrafish larvae at 2 dpf were treated with PS or PTS (400 μg/mL) and arbutin (1 mM) for 48 h, and images were collected (left four zebrafish larvae). Additionally, 2 dpf zebrafish larvae were treated with PTU (200 μM) for 24 h and α-MSH (1 μg/mL) was treated for another 24 h. Next, the larvae were treated with PS or PTS (400 μg/mL) and arbutin (1 mM) for 48 h. The effect of PS, PTS, and arbutin on pigmentation in zebrafish larvae was observed

under a microscope (40×). Relative density was calculated by Image J software. The percentage values in each group are relative to those in untreated group. Data are reported as the mean ±SE of three independent experiments performed ($n = 3$). $^{###}$ $p < 0.001$ *vs*. untreated group; *** $p < 0.001$ *vs*. α-MSH-stimulated group in B16F10 cells (A). $^{#}$ $p < 0.05$, $^{##}$ $p < 0.01$, and $^{###}$ $p < 0.001$ *vs*. untreated control group; *** $p < 0.001$ *vs*. α-MSH-stimulated group pretreated with PTU in zebrafish larvae (B).

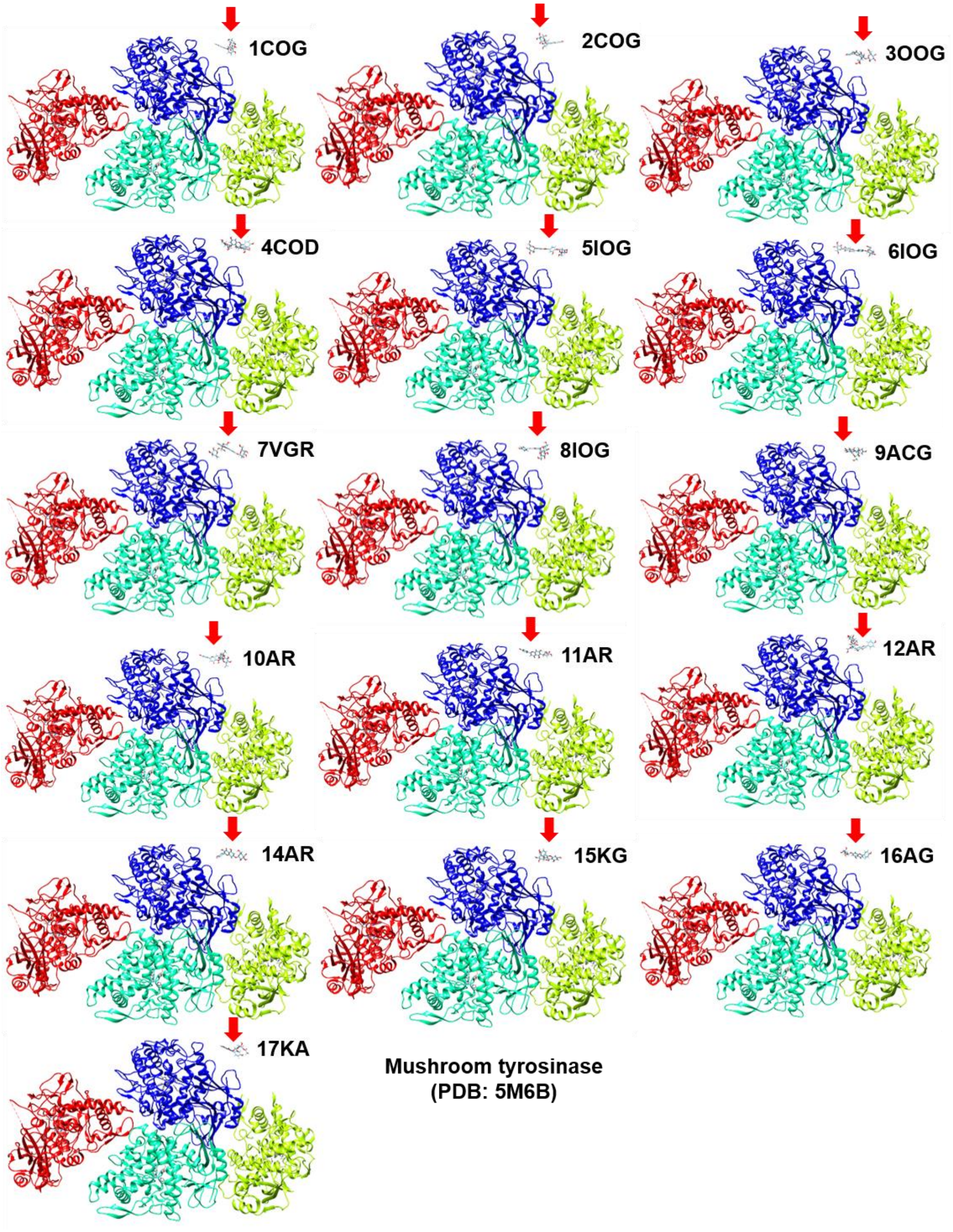

Figure S4. Molecular docking of mushroom tyrosinase (PDB: 5M6B) and anthocyanins confirmed in PS and PTS. Anthocyanins do not directly bind to mushroom.

Table S1. Classification of results gained from the docking of anthocyanins identified in this study into DUSP7.

Receptor	Anthocyanins	Docking score*
DUSP7 (2Y2E)	Cyanidin-3-*O*-galactoside	-6.2
	Cyanidin-3-*O*-glucoside	-6.8
	Orientin-7-*O*-glucoside	-5.7
	Cyanidin-3,5-*O*-diglucoside	-6.3
	Isoorientinm-4'-*O*-glucoside	-6.8
	Isovitexin-4'-*O*-glucoside	-6.7
	Vitexin-4'-*O*-glucoside-2''-*O*-rhamnoside	-7.3
	Isovitexin-7-*O*-glucoside (saponarin)	-5.5
	Apigenin-8-C-β-D-glucopyranoside (Vitexin)	-6.0
	Isovitexin-2''-*O*-rhamnoside	-6.2
	Apigenin-6-C-β-D-glucopyranoside (Isovitexin)	-6.0
	Apigenin-6-C-glucoside-7-(6''-*O*-acetyl)-glucoside	-7.2
	Kaempferol-*O*-glucoside derivative	**
	Kaempferol-7-*O*-glucoside	-7.3
	Kaempferol-3-*O*-glucoside	-5.8
	Apigenin-7-*O*-glucoside	-7.4
	Kaempferol-3-(6''-acetylglucoside)	-6.6

*, Docking score are highest one from 4 different docking pose.

**, Chemical structure was not found from PubChem CID.
